# Right Place, Right Time: Spatiotemporal Predictions Guide Attention in Dynamic Visual Search

**DOI:** 10.1037/xge0000901

**Published:** 2021-11-29

**Authors:** Sage E. P. Boettcher, Nir Shalev, Jeremy M. Wolfe, Anna C. Nobre

**Affiliations:** 1 Department of Experimental Psychology, University of Oxford; 2 Oxford Centre for Human Brain Activity, Wellcome Centre for Integrative Neuroimaging, Department of Psychiatry, University of Oxford; 3 Visual Attention Laboratory, Department of Surgery, Brigham and Women’s Hospital, Boston, Massachusetts, United States; 4 Harvard Medical School

**Keywords:** visual search, temporal attention, spatial attention, memory

## Abstract

Visual search is a fundamental element of human behavior and is predominantly studied in a laboratory setting using static displays. However, real-life search is often an extended process taking place in dynamic environments. We have designed a dynamic-search task in order to incorporate the temporal dimension into visual search. Using this task, we tested how participants learn and utilize spatiotemporal regularities embedded within the environment to guide performance. Participants searched for eight instances of a target that faded in and out of a display containing similarly transient distractors. In each trial, four of the eight targets appeared in a temporally predictable fashion with one target appearing in each of four spatially separated quadrants. The other four targets were spatially and temporally unpredictable. Participants’ performance was significantly better for spatiotemporally predictable compared to unpredictable targets (Experiments 1–4). The effects were reliable over different patterns of spatiotemporal predictability (Experiment 2) and primarily reflected long-term learning over trials (Experiments 3, 4), although single-trial priming effects also contributed (Experiment 4). Eye-movement recordings (Experiment 1) revealed that spatiotemporal regularities guide attention proactively and dynamically. Taken together, our results show that regularities across both space and time can guide visual search and this guidance can primarily be attributed to robust long-term representations of these regularities.

Our environment is filled with regularities that can guide our perception and facilitate performance (Nobre & van Ede, 2018; Võ et al., 2019). As we prepare an afternoon cup of tea, our prior experience allows us to orient attention to the kettle the moment before it whistles and then to a spoon stored away in the top drawer. Such attentional guidance is particularly beneficial when multiple signals compete for our attention; for instance, if the kettle is placed in a messy kitchen and loud music is preventing us from hearing it. In cognitive research, a popular choice for studying attention in the context of competition and distraction is the visual search task (Treisman & Gelade, 1980; Wolfe, 2020, 2021). In such tasks, observers search for a target among distractors in a visual display. There is mounting evidence that regularities learned over various timescales significantly benefit performance and that performance in search tasks drastically improves when regularities learned from varying time scales can be exploited (Awh et al., 2012; Chun & Jiang, 1998, 2003; Jiang et al., 2005; Kristjánsson & Campana, 2010; Maljkovic & Nakayama, 1994; Nobre & Stokes, 2019).

It is now relatively established that various task properties can influence where or what people attend over short time scales. A clear example comes in the form of “repetition priming” whereby performance is facilitated when target items share features (Campana et al., 2008; Kristjánsson, 2006; Maljkovic & Nakayama, 1994; Meeter & Olivers, 2006) or locations (Maljkovic & Nakayama, 1996; for review see Kristjánsson & Campana, 2010) with items that were recently selected. For example, Maljkovic and Nakayama (1996) showed facilitation when the target position was repeated from the previous trial and inhibition when the target appeared in the same location as a distractor. Studies that specifically compared the benefits of explicit cues and repetition priming found that these effects differed in their magnitude and duration suggesting different underlying mechanisms between the two processes (Maljkovic & Nakayama, 2000). As such, priming is generally considered to be a product of an implicit short-term memory (STM) system (Becker, 2008; Kristjánsson & Campana, 2010; Maljkovic & Nakayama, 2000; Schacter & Buckner, 1998; Sigurdardottir et al., 2008).

Learning over longer time scales (i.e., beyond a single-trial repetition) also contributes to the allocation of attention. For example, in probability-cuing tasks observers search for a target in an environment with high-probability target locations, and low-probability target locations. Observers are faster at finding targets that occur in the high-probability locations (Geng & Behrmann, 2002, 2005; Jiang, 2018; Jiang, Swallow, Rosenbaum & Herzig, 2013; Shaw & Shaw, 1977). This effect cannot be explained by repetition priming alone (Goschy et al., 2014; Jiang, Swallow, & Rosenbaum, 2013; Jones & Kaschak, 2012). Contextual cuing benefits in visual search task also clearly suggest the impact of longer-term memories in guiding attention. In contextual cuing tasks, performance benefits result when a target appears at the same location within a given configuration of distractors that reoccurs (Chun & Jiang, 1998). The effects are independent of the immediate repetition of target locations between successive trials and have even been shown after a delay of 1 week (Chun & Jiang, 2003).

Taken together, the facilitation of performance based on short- and long-term representations in visual search has been conceptualized within statistical-learning and selection-history frameworks (Awh et al., 2012; Theeuwes, 2019). The central idea is that previous attentional deployments can have lasting biasing effects that are independent of task goals or physical salience. These effects are typically fast acting, automatic, flexible, and have been shown not to depend on the participants’ awareness of the environmental regularities (Chun & Jiang, 1998, 1999; Goujon et al., 2015). Furthermore, studies investigating visual search within natural scenes also reveal the contribution of semantic and grammatical (structural) associations as additional long-term memory sources of attentional guidance (e.g., kettles are usually found on kitchen counters, not bathroom floors), (Võ et al., 2019; Wolfe et al., 2011). Such theoretical notions and findings fit within the broader proposal that the contents of memories of different types and timescales guide perception by influencing the deployment of attention (Nobre & Stokes, 2019).

Visual search studies have provided some of the cornerstones to our understanding of how attention is controlled and guided. To date, most studies only investigate search within discrete perceptual events, such as a briefly presented static display or scene. However, search in the real world occurs within an unfolding temporal context. For example, if you are searching for a friend at a crowded train station. Searching for target items among dynamic sets of distractors in unfolding temporal contexts introduces additional challenges in terms of sustaining optimal performance and learning about useful regularities. Regularities between items often occur across temporal intervals that are filled with distraction. To be effective, therefore, spatial or identity-related predictions should also carry temporal information (e.g., when your friend is likely to arrive). Furthermore, extracting regularities about the timing of relevant events occurring among distracting stimuli requires more than learning about simple associations about the order or the temporal interval between individual events. Instead, the timing of target items must be abstracted across the entire duration of irrelevant distracting events if it is to aid search based on item identity or location.

Thus, investigating the guidance of attention within temporally extended contexts provides an important next step toward our understanding of the mechanisms contributing to real-life visual search. A few different experimental approaches have started to examine the temporal dimension within visual search. For example, researchers have shown that a regularly occurring sequence of events can attract attention automatically without the need of prior experiences (Zhao et al., 2013). Moreover, recent work has also demonstrated that people can use a continuous change of features (e.g., gradual change of color or change of number) within a dynamic task to anticipate when a stimulus becomes response relevant (Muhl-Richardson, Cornes, et al., 2018; Muhl-Richardson, Godwin, et al. 2018). Li and Theeuwes (2020) demonstrated that observers anticipate targets based on regular patterns of target sequences (e.g., they can learn that the selection of a target at one location the visual field is likely to be followed by a target at another location). These studies establish that predictions based on sequential associations between successive stimuli (or gradual modulations of stimuli features) can be learned and utilized to improve performance.

Other studies have suggested that learned associations about the temporal interval between events, and not merely their temporal order, can also be used to improve search performance. Building on the literature of temporal orienting of attention, or temporal expectation (see Nobre & Rohenkohl, 2014; Nobre & van Ede, 2018, for reviews), studies have shown performance benefits for targets occurring at temporally predictable intervals within simple arrays. Olson and Chun (2001) attempted to understand temporal orienting on the basis of long-term memories by manipulating the intervals used in the sequential structure between visual events. When this temporal context could be implicitly learned, observers were able to orient their attention in both time and space. Cravo et al. (2017) showed that the timing of an event within a natural scene could be learned to trigger neural signatures of temporal anticipation and performance benefits. Additionally, work investigating temporal associations has highlighted how expectations regarding the timing of stimuli can interact strongly with spatial expectations (Doherty et al., 2005; Heideman et al., 2018; Nobre & Rohenkohl, 2014; O’Reilly et al., 2008; Rohenkohl et al., 2014).

Taken together, these studies provide a promising foundation for investigating visual search in temporally extended and noisy contexts. Building on this work, we have developed a new experimental approach to ask whether and how spatiotemporal predictions about the locations and timings of relevant items can be extracted within noisy dynamic contexts to guide attention and improve search performance. Specifically, our aim was to answer two important questions within the field: (a) Does what we have learned about regularities in static visual search displays apply to dynamic displays? (b) Can we learn and use regularities regarding the temporal onsets of stimuli appearing in a context of other, temporally unpredictable distractors? Such a finding would require the ability to learn temporal markers for stimuli that are cannot be reduced to simple temporal order (e.g., the 3rd stimulus is likely to appear in this location) or derived by learning the intervals between individual successive events.

In our “dynamic visual search” task, targets and distractors unfold over time at various spatial locations, allowing us to test whether learned regularities continue to guide performance in the face of intervening distracting events distributed in time and space. Benefits of performance would require abstraction of predictable locations and timings of relevant stimuli embedded within the context of other dynamically unfolding, but irrelevant stimuli. This approach introduces an important step change from previous studies that have considered the effects of simple associations about the serial order or timing between successive events. Within an extended context with unpredictable intervening distractors, learning simple associations between successive stimuli would be insufficient for exploiting the spatial and temporal regularities in our stimuli.

Our experimental approach consists of a visual-search task in which targets and distractors appear and disappear within a noisy background over the course of several seconds. Observers are asked to find multiple instances of a target (vertical line) and to ignore distractors (tilted lines). On each trial, half of the vertical targets appear at predictable times and locations, while the other half of the targets appear completely unpredictably. Across four experiments, we show superior behavioral performance for identifying the targets that appear at consistent locations and times during these dynamic trials (Experiments 1–4). We show that the effects are robust across different arrangements of spatiotemporal regularities (Experiment 2). They are not dependent on the immediate repetition of spatiotemporal predictions between trials (Experiment 3), though they did benefit modestly from repeating patterns between successive trials (Experiment 4). We believe our results provide strong and reliable evidence for the ability to utilize spatiotemporal regularities to guide search in extended dynamic contexts, such as those present in our daily interactions. While far from providing a definitive account of how dynamic search happens, our experimental approach provides a flexible and promising experimental platform that can be used to investigate how regularities along multiple stimulus dimensions (e.g., identifying features, locations, time points, response associations) contribute and interact to prioritize, anticipate, and select relevant items and overcome distraction to guide adaptive behavior.

## Experiment 1: Spatiotemporal Regularities in Dynamic Visual Search Guide Behaviour

### Method

#### Participants

We tested 25 participants (age range 18–30, *M_age_* = 23.4, 11 females). All participants had normal or corrected-to-normal vision, provided written consent, and were compensated at a rate of £10 per hour.

We chose our sample size based on the large effect sizes in the probability and contextual cuing and statistical learning literature (Chun & Jiang, 1998; Jiang, 2018; Jiang, Swallow, & Rosenbaum, 2013; Li & Theeuwes, 2020) and on preliminary piloting efforts. For completeness, we ran a posthoc simulation-based power analysis on the results from Experiment 1 using the mixedPower Package in R (Kumle et al., 2021). Since posthoc procedures can overestimate true effect sizes, we conducted a simulation using half of the effect size observed in Experiment 1. We found that a sample size of 25 participants would lead to a power above 80% for detecting an effect that is half as large as the one we observed in the data.

#### Apparatus

Participants were positioned on a chin rest 100 cm from the monitor (22-in. Samsung SyncMaster 2233; resolution 1,680 × 1,050 pixels; refresh rate 100 Hz; screen width 47 cm). Eye movements were recorded with the Eyelink-1000-plus desktop mount (SR Research, Ontario, Canada) at 1,000 Hz. A 9-point calibration was used with an error threshold of .5° visual angle. Observers who did not meet this calibration threshold were not included in the final eye-track analysis, leaving 20 participants for this analysis. Drift correction was applied between blocks. The experimental script was generated using Psychophysics Toolbox (Brainard, 1997) on MATLAB (Version 2014b, Mathworks, Natick, MA).

#### Stimuli

The search display consisted of a 2 × 2 array of four different 1/F static noise patches that were generated for each trial. Each quadrant extended approximately 8.3° (horizontal angle) × 5.8° (vertical angle). Forty distractor stimuli (tilted grey bars, 80°–100°, RGB values [88,88,88]) and eight target stimuli (vertical grey bars) appeared and disappeared at different times over the course of approximately 14 second trials. These bars did not move in their location, but rather faded slowly in and out of view. For all stimuli, the fade-in time was 2 seconds (gradually becoming visible over 80 refresh-rate cycles until reaching maximum visibility). The stimulus then remained at maximum contrast for another .8 seconds and faded out over 2 seconds. For purposes of analysis, onset times were defined as the first moment from when stimuli started fading in. This differs from the time at which the stimulus becomes subjectively detectable to the participant over which we did not have control. Total stimulus duration was 4.8 seconds. Each stimulus was ∼.5° in length and ∼.08° in width and could appear anywhere within the boundaries of one of the four quadrants as long as it did not overlap with another stimulus. Because they were presented on 1/F noise, the visibility of items varied unpredictably; however, any difference in visibility was unrelated to the status of the item as a predictable or unpredictable target or distractor.

#### Procedure

**Figure 1 fig1:**
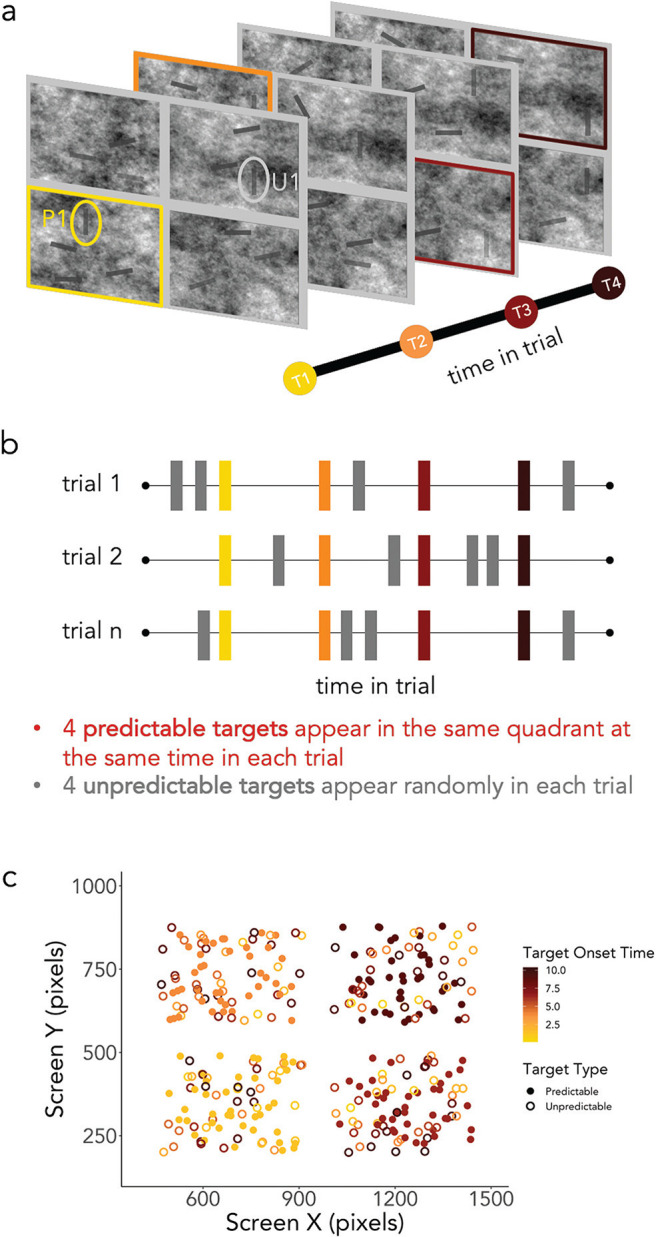
Trial Schematic for Experiment 1 *Note*. (a) On each trial, participants searched for eight vertical bars among distractors—a yellow (gray) circle indicates the first predictable (unpredictable) target. Targets and distractors appeared and disappeared over the course of the trial. (b) The time course of a trial is depicted with the onset of trial events represented as rectangles (colored rectangles represent predictable targets while gray rectangles represent unpredictable targets. (c) Here is an example of a single participant’s target events across one block of trials. Predictable targets are represented as filled circles whereas unpredictable targets are represented as unfilled circles. The time within a trial is represented from yellow (light gray) to dark red (dark gray). Here it can be seen that predictable targets always appear at the same time and within the same quadrant (although the exact location within the quadrant varied). Unpredictable targets had variable onsets and could appear anywhere. See the online article for the color version of this figure.

All experimental procedures were reviewed and approved by the Central University Research Ethics Committee of the University of Oxford. Observers were instructed to find and click on eight small gray vertical lines which appeared and disappeared over the course of a trial (Figure 1a). Of the eight target stimuli, four were predictable. They appeared at the same time relative to the start of the trial and in the same quadrant on every trial (Figure 1b). Their specific location within the quadrant was random (Figure 1c). Thus, it would be possible to learn that, at time X, a target will always appear somewhere in quadrant Y. These predictable targets appeared every 2.5 seconds, with the onset of the first target occurring 1.25 seconds into the trial.[Fig fig1]

Forty-four other items were presented during each trial. We uniformly distributed the onset of these items across the time in the trial by pseudorandomly choosing a start time when each item began fading in. This distribution was constrained to present 11 of the 44 items within each successive 2.5 second window (thus creating 4 artificial and seamless time bins within the trial). Once the 44 items were assigned a particular starting time, we chose four of those items to serve as the unpredictable targets. This assignment was not contingent on the temporal distance between the unpredictable targets or the distance to the predictable targets. This means that the exact temporal relations between distractors and unpredictable and predictable targets were not predetermined or constrained. All stimuli had the same temporal profile, and a trial ended when the last stimulus had completely faded out of the display. The spatial locations of the distractors and unpredictable targets were determined randomly. For each item we randomly chose a quadrant (1–4) and then within that quadrant randomly chose a spatial location with the only constraints being that the items should not overlap or cross the quadrant border. Note that this means that the number of unpredictable targets in each quadrant was unpredictable on any given trial. The task is depicted in Figure 1 and a sample trial can be found in https://osf.io/py2w4/ (note the green dots in the video are for visualizing responses and did not actually appear in the experiment).

Observers completed four blocks of 40 trials. Trials were terminated when the last stimulus had fully disappeared from the display. This time varied slightly across trials as the onsets of unpredictable targets and distractors were chosen randomly. Each trial contained eight targets (4 *predictable* and 4 *unpredictable*). Overall, there were 8 × 4 × 40 = 1,280 target events per participant. After each trial, observers received feedback in the form of a number between 0 and 8 indicating how many targets they had found. They were able to proceed at their own pace. The experiment lasted approximately 45 minutes.

#### Behavioral Analysis

Behavioral data were analyzed using R (R Core Team, 2018) using the approach described in Helbing et al. (2020) and Draschkow and Võ (2017). Differences in analysis procedures between experiments are highlighted in the corresponding section. Within each experiment, participants with performance below 2 standard deviations (*SD*) from the mean performance of all subjects were discarded. For the remaining observers, responses with RTs above or below 3 *SD* of the mean were discarded. This resulted in an average loss of fewer than .05% of trials across all four experiments.

For each experiment there were two dependent variables of interest: accuracy and reaction time (RT). Accuracy was defined as the percentage of total targets found (e.g., accuracy of 80% would mean an observer found on 80% of the potential targets). RT was defined as the time between the target onset (i.e., the moment a target began to fade in) and the time an observer clicked on a target. These RTs are more complicated than those from a static visual search task. RT will depend on visibility, and visibility of a specific target will depend on many factors including the random noise background beneath and surrounding the target and where an observer happened to be fixating when the target began to appear. In addition, cursor position at the time of target detection will also contribute to the overall RT. While these factors introduce noise into the RT measure, they do not differ systematically between the conditions. As such, the extra noise within this measure does not compromise any comparisons of performance between predictable and unpredictable targets. That is, if predictable targets systematically attract attention before unpredictable targets, this will be clear from the RTs.

Generalized linear mixed-effects models (GLMMs) with a binomial distribution were used to analyze accuracy as a binary response and linear mixed-effects models (LMMs) were used to analyze the reaction times for correct trials in all experiments. Each row of the model contained a single target event. With eight targets per trial, 40 trials per block, and four blocks per participant there were 1,280 target events per participant. Because RT analysis was restricted to correct trials, there were fewer trial events in the LMMs. These analyses were run using the lme4 package (Version 1.1–17; Bates et al., 2015). We used mixed-effects models as they hold multiple benefits over a more traditional approach to analysis of variance. Importantly for the current study, these approaches are more reliable in unbalanced designs when different conditions may have different trial numbers (this becomes critical Experiments 3 and 4; Baayen et al., 2008). All GLMMs and LMMs were fitted with the maximum likelihood criterion. For the GLMMs, where we report regression coefficients β with the z statistic and use a two-tailed 5% error criterion for significance, the *p*-values for the binary accuracy variable are based on asymptotic Wald tests. For the LMMs, we report β with the *t*-statistic and apply a two-tailed criterion corresponding to a 5% error criterion for significance. The *p*-values were calculated with Satterthwaite’s degrees of freedom method using the lmerTest package (Version 3.1-0; Kuznetsova et al., 2017). The ggplot2 package (Version 3.1.0; Wickham, 2009) was used for plotting. In addition, to benchmark against studies using more traditional analysis approaches, we conducted repeated-measures analyses of variance (ANOVAs). These showed equivalent results and can be found in Tables S1 and S2 in the online supplementary materials as well as in the analysis script (Boettcher & Shalev, 2021), which can be found in https://osf.io/8avtq/.

In Experiment 1, there were two main independent variables of interest: predictability (Predictable vs. Unpredictable) and, within the levels of predictability, target order (1st, 2nd, 3rd, or 4th). Target order was centered and entered the model as a continuous predictor. The critical comparison between predictable and unpredictable targets was modeled using sum contrasts (with predictable targets being coded as 1 and unpredictable targets coded as −1). As such the grand mean of the dependent measure served as the intercept. For binary responses such as accuracy in the GLMM approach, the coefficients were represented by logits. We began each model with a maximal random-effects structure (Barr et al., 2013) that included intercepts for each participant, as well as by-participant slopes for the effects of Target Order and Predictability. Full models such as these often fail to converge or lead to overparameterization (Bates et al., 2015). Therefore, we used a principal component analysis (PCA) of the random-effects variance-covariance estimates to identify overparameterization for each fitted model and removed random slopes that were not supported by the PCA and did not contribute significantly to the goodness of fit in a likelihood ratio (LR) test (Bates et al., 2015). In Experiment 1, the GLMM’s random-effects structure contained the subject intercepts as well as by-subject slopes for predictability and target order (i.e., the full model). The optimal LMM for predicting participants’ reaction times contained the participant intercepts as well as the participant slopes for target order. Pairwise tests following significant interactions were further investigated using the lsmeans package (Lenth, 2016) with Tukey posthoc correction. Further details regarding the models and model comparisons can be found in the analysis script (Boettcher & Shalev, 2021) available in https://osf.io/8avtq/.

#### Eye Tracking Analysis

Of the 25 participants, 20 had usable eye-tracking data. The raw eye-position samples for these 20 participants were first converted to a data matrix using a Matlab script. The raw data matrix contained the X-Y coordinates of gaze position (in pixel units) throughout the task. Data were then recoded to a single vector of spatial quadrant (i.e., each sample of X and Y coordinates was combined and recoded to a single number between 1 and 4, representing the spatial quadrant at which eyes were fixated at any given moment). This conversion from screen coordinates to quadrants was important for two key reasons. First, the spatial predictions formed in this experiment were on the level of the quadrant rather than at the specific position of a target (see Figure 1) and therefore we were primarily interested in how these quadrant-based predictions affected eye movements. Second, due to the rather small size of our stimuli, the noise of the background, and the dynamically changing opacity of the stimuli, it would have proven quite difficult to define interest areas that accurately reflected the targets’ visibility. The time series was then converted to a probability matrix for each quadrant separately and split by trials to allow statistical analysis.

To quantify spatiotemporal predictions, we compared the mean probability of searching within a quadrant with a predictable versus unpredictable target, within a time window of 4.5 seconds, commencing 500 ms before stimulus onset. The comparison relied on a permutation test based on 5,000 samples. The resulting distribution for each data point was compared to a critical *t*-value (*p* < .05) corrected for multiple comparisons based on the “t-max” method (Blair & Karniski, 1993; Westfall et al., 1993).

### Results and Interim Discussion

**Figure 2 fig2:**
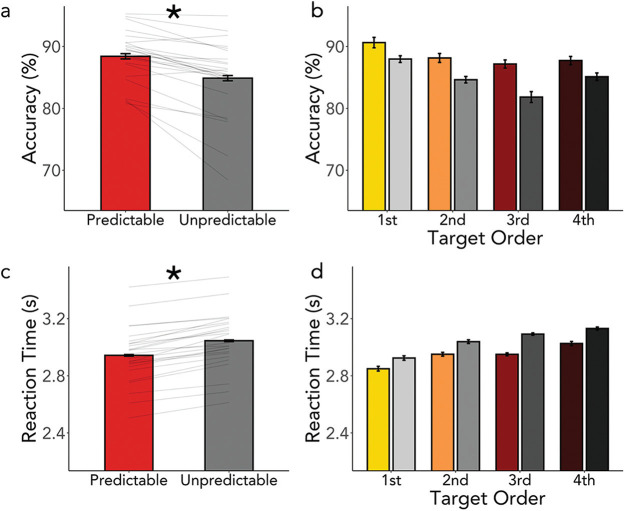
Spatiotemporal Regularities in Dynamic Visual Search Guide Behavior *Note*. (a) Mean accuracy for predictable and unpredictable targets. (b) Mean accuracy across the trial. Colors follow the convention in Figure 1. (c) Mean RT for predictable and unpredictable targets. (d) Mean RT across the trial. Error bars in bar graphs represent the standard error of the mean, individual participants are represented as light gray lines, and stars indicate a significant difference with a *p*-value < .05 in this and all subsequent figures. See the online article for the color version of this figure.

**Figure 3 fig3:**
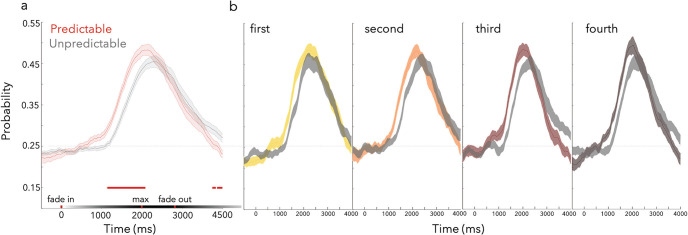
Gaze is Biased Towards Predictable Targets Earlier *Note*. (a) The probability of a fixation landing in the “target” quadrant for a 5-second epoch around the “onset” of the target (Time 0). The target dynamics are indicated on the x axis and the moment fade in begins (0s), the moment max opacity is reached (2 s), and when the fade out begins (2.8 s) are labeled as well. Note the shading and color of this line is illustrative and does not actually reflect the color values of the target. Given there are four quadrants, the chance of being in any one quadrant would be .25. The onset indicates the moment that the targets were no longer completely transparent, although they were not necessarily visible at this moment and shaded areas around the line represent the 95% confidence interval. The probability of fixating a target quadrant was significantly higher for predictable targets early in the epoch and significantly lower during the later stage of the epoch. Significant time windows are marked with a solid red (black) line. (b) This pattern repeats throughout the different instances of the target. See the online article for the color version of this figure.

Performance on the task is shown in Figures 2a–d and shows that observers were more accurate and faster to respond to targets that were spatiotemporally predictable. Eye-movements analysis, shown in Figures 3a–b, reveals that observers were more likely to fixate the target quadrant earlier for predictable compared to unpredictable targets.[Fig fig2][Fig fig3]

#### Accuracy

In Experiment 1, we found that participants were significantly more likely to identify a predictable compared to an unpredictable target (Figure 2a; β = .15, *SE* = .02, *z* = 6.95, *p* < .001). Additionally, we found a main effect of the target order such that observers were better at detecting earlier targets (Figure 2b; β = −.09, *SE* = .02, *z* = −4.50, *p* < .001). However, there was no significant interaction between target predictability and target order, indicating the effect of predictability was present throughout the course of the trial (β = −.003, *SE* = .02, *z* = −.227, *p* = .82). The individual participants’ results in each of the four experiments are plotted in Figure S1 in the online supplementary materials. Specifically, the effect of predictability on accuracy (predictable–unpredictable targets is shown across target order. The vast majority of observers showed higher accuracy for predictable compared to unpredictable targets across all targets within a trial.

#### RTs

Participants were significantly faster at finding predictable targets compared to unpredictable targets (Figure 2c; β = −.052, *SE* = .004, *t* = −12.23, *p* < .001), as well as, significantly faster at finding targets appearing early in the trial compared to late targets (Figure 2d; β = .062, *SE* = .005, *t* = 12.87, *p* < .001). These two factors showed a small yet significant interaction (β = −.007, *SE* = .004, *t* = −1.96, *p* = .049). However, posthoc comparisons showed significant differences between predictable and unpredictable targets at each point (*z*s > 5.8, *p*s < .001). Again, individual participants’ effects are included in Figure S1 in the online supplementary materials.

In Experiment 1, we also recorded eye-movement data to investigate whether spatiotemporal predictions influenced the pattern of eye movements. We compared the probability of looking at the quadrant in which predictable versus unpredictable targets were presented at each time point locked to the onset of a target (500 ms before onset until 4500 ms after). The results, illustrated in Figure 3a, shows a higher probability to fixate the target quadrant for predictable compared to unpredictable targets early in the trial beginning at approximately 1,100 ms and lasting for ∼1,000 ms. The difference was driven by the higher probability to fixate at the target quadrant when searching for a predictable target. A significant change in fixation probability also occurred at a later time window (∼4,250 ms), this time showing a smaller likelihood of fixating the quadrant in which a predictable target occurred. The lower rates of fixation may reflect a faster general spatial disengagement after quicker detection of predictable versus unpredictable targets or it may also reflect the inferred lower likelihood for the next target to occur in the same quadrant. Figure 3b makes clear that this pattern of fixation probability was present throughout the trial.

## Experiment 2: Behavioral Benefits Hold Up Against Asynchronous Regularities

Experiment 1 shows that observers were more accurate and faster at finding spatiotemporally predictable compared to unpredictable targets, and this is true throughout the trial. Experiment 2 was aimed to reduce effects in Experiment 1 that could be attributed to the strictly even distribution of predictable targets. We varied the timing of predictable targets across participants, such that the timing of onsets was consistent for any given participant, but those timings were not necessarily evenly distributed across the course of a trial. If the effects of target predictability are still present, then it is clear that these results cannot be solely attributed to a rhythmic pattern of attentional allocation.

### Method

#### Participants

Twenty-five participants took part in Experiment 2. One participant was discarded for low performance—with an average accuracy more than 2 *SD* away from the mean. The remaining 24 participants were between 18 and 30 years old with an average age of 22.83. The sample contained 20 females. All participants had normal or corrected-to-normal vision, provided written consent, and were compensated at a rate of £10 per hour.

#### Apparatus

Participants completed the experiment in a group testing room with a capacity of 20 people, although no more than 12 were tested at once. Participants each sat approximately 60 cm from the monitor (Dell U2312HM Monitor, 1,920 × 1,080 resolution; refresh rate 60 Hz). The experimental script was again generated using the Psychophysics Toolbox (Brainard, 1997) on MATLAB (Version 2014b, Mathworks, Natick, MA).

#### Stimuli

In Experiments 2–4 the timings of the stimuli were changed slightly such that the speed of the fading was increased slightly. Specifically, each stimulus faded in over 1.3 seconds (gradually becoming visible over 80 refresh-rate cycles until reaching maximum visibility). Then the target stayed on the screen for another 1.3 seconds and faded out over 1.3 seconds. The search display again consisted of four unique 1/F static noise patches that were generated for each trial. Each quadrant extended approximately 12.6° (horizontal angle) × 8.84° (vertical angle). Each stimulus was ∼.75° in length and ∼.13° in width and could appear anywhere within the boundaries of one of the four quadrants as long as it did not overlap with another stimulus.

#### Procedure

**Figure 4 fig4:**
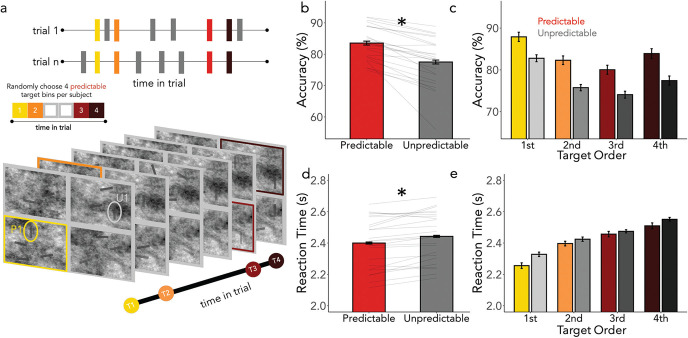
Increased and Faster Detection of Predictable Targets With Asynchronous Regularities *Note*. (a) The trial schematic for Experiment 2. Each trial was divided into six time bins, and each observer was randomly assigned to four bins—a yellow (gray) circle indicates the first predictable (unpredictable) target. (b) Mean accuracy is plotted for predictable and unpredictable targets—individual participants are represented as light gray lines. (c) Mean accuracy is plotted across the trial split by predictability. Mean RT for predictable and unpredictable targets (d) averaged over the trial and (e) separately for target across the trial. Asterisks indicate a *p*-value < .05. See the online article for the color version of this figure.

The procedure was nearly the same as in Experiment 1, but a few changes were introduced. In Experiment 2, six (instead of four) sequential time windows were used to determine the onset time for each stimulus and to ensure the onsets were evenly distributed throughout the trial. To determine the onset of the predictable targets, four of the six time bins were randomly selected for each observer and the temporal midpoints of these bins were used. Thus, the appearance of targets was predictable but did not follow regular intervals. The quadrants and specific locations of the unpredictable targets were chosen randomly. Unpredictable targets could appear at any moment throughout the trial and their onsets were not constrained to occur during the time bins used for the four predictable targets. The rest of the experimental procedures were the same as in Experiment 1. The experimental task is depicted in Figure 4a.[Fig fig4]

#### Behavioral Analysis

We followed the same analysis procedure as in Experiment 1. In Experiment 2, the random-effects structure of the GLMM contained the participant intercepts as well as by-participant slopes for predictability and target order (i.e., the full model). The full model was also optimal for the LMM in Experiment 2.

### Results and Interim Discussion

Results are summarized in Figure 4b–e and showed that the benefits of predictability were preserved even when the predictable targets did not appear at evenly spaced moments in time.

#### Accuracy

As in Experiment 1, there was a main effect of predictability on the accuracy, with predictable targets being found significantly more often than unpredictable targets (Figure 4b; β = .19, *SE* = .02, *z* = 8.59, *p* < .001). We once again found a main effect of target order on accuracy with higher accuracy for early targets (Figure 4c; β = −.10, *SE* = .02, *z* = −4.52, *p* < .001), and the interaction was again not significant (β = −.0005*; SE* = .01, *z* = −.045, *p* = .96).

#### RTs

In a replication of Experiment 1, an analysis of RTs revealed a main effect of predictability (β = −.02, *SE* = .004, *t* = −4.72, *p* < .001) as well as target order (β = .08, *SE* = .005, *t* = 14.35, *p* < .001). More specifically, observers were faster for predictable targets (Figure 4d) as well as targets appearing early in the trial (Figure 4e). Again, the interaction was not significant (β = .004, *SE* = .003, *t* = 1.53, *p* = .13).

## Experiment 3: Spatiotemporal Guidance Is Robust Against Single-Trial Interference

In Experiments 1 and 2 we established that regularities in time and space can help guide dynamic visual search. In both of these experiments there were two major sources of regularities, either or both of which may have contributed to performance facilitation: short-term priming effects from one trial to the next and the build-up of longer-term memories related to regularities extracted over the course of the experiment. Although these sources of information are highly related, we wished to distinguish, to the extent possible, the relative contributions of single-trial priming and longer-term learning. To do so, in Experiment 3, we asked whether single-trial priming effects could fully explain the behavioral benefits of target predictability. If this were the case, one would expect that the performance benefits in predictable versus unpredictable targets would be eradicated following a trial that contained no regularities. Therefore, in Experiment 3 we introduced a new trial type that did not contain any predictable targets (random trials). If performance benefits are fully reliant on single-trial priming, then these benefits should disappear on trials immediately following a random trial.

### Method

#### Participants

Twenty-seven observers were tested. One participant had an average accuracy more than 2 *SD* lower than the mean, leaving a final sample size of 26 (age range 18–33, mean age = 24.5, 20 females). All participants had normal or corrected-to-normal vision, provided written consent, and were compensated at a rate of £10 per hour. The group of participants in this experiment also participated in Experiment 4 (see below) within a single session.

#### Apparatus

Participants completed the experiment in a group testing room with a capacity of 20 people, although no more than 12 were tested at once. Participants each sat approximately 50 cm from the monitor (Dell U2312HM Monitor, 1,920 × 1,080 resolution; refresh rate 60 Hz). The experimental script was again generated using the Psychophysics Toolbox (Brainard, 1997) on MATLAB (version 2014b, Mathworks, Natick, MA).

#### Stimuli

The stimuli were the same as in Experiment 2.

#### Procedure

The group that participated in this experiment also completed a second experiment (Experiment 4; see below), and the order of task administration was counterbalanced.

**Figure 5 fig5:**
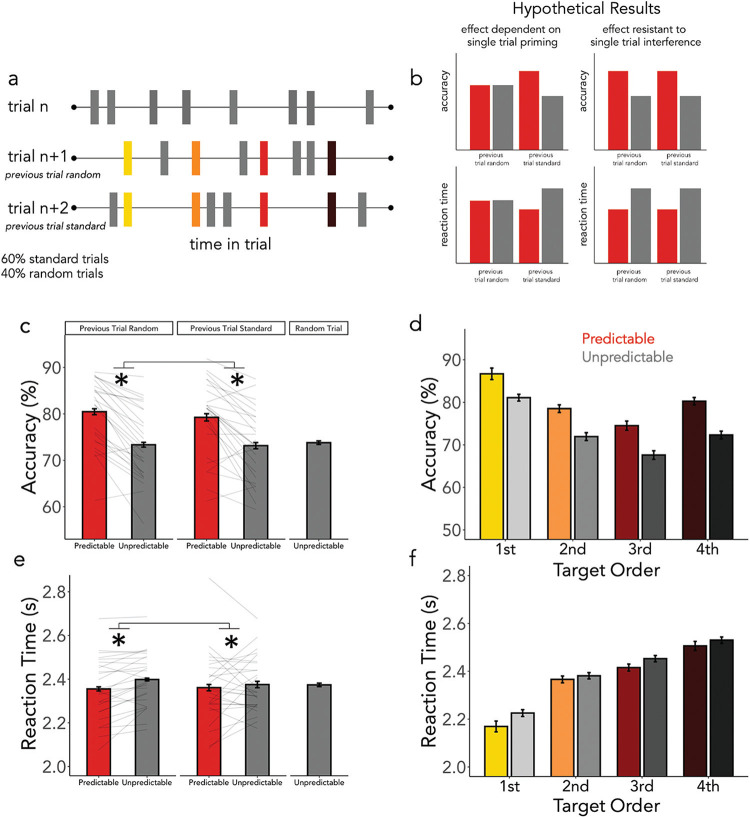
Spatiotemporal Guidance Is Held in Long-Term Memory *Note*. (a) The trial schematic for Experiment 3. In 40% of trials, the locations and timings of all targets were completely unpredictable. (b) Hypothetical results are shown for two scenarios: the predictability effect is dependent on single-trial priming (left) or the predictability effect is resistant to interference (right). (c) Mean accuracy is plotted for predictable and unpredictable targets separated by the previous trial type—individual participants are represented as light gray lines. Participant means for the average accuracy in fully random trials are also shown. (d) Accuracy in standard trials is plotted across the trial. (e) Mean RTs are plotted for predictable and unpredictable targets when the target was preceded by a standard trial, a random trial, as well as for when the trial itself was fully random. (f) The reaction times in standard trials across the entire trial. Asterisks indicate a *p*-value < .05. See the online article for the color version of this figure.

Sixty percent of the trials were standard trials as described in Experiment 1. However, in 40% of the trials all targets were completely unpredictable. That is, in these trials, the four predictable targets appeared at an unpredictable time and quadrant. Trial order was arranged such that random trials were always followed by standard trials. This constraint enabled us to test whether the benefit of the spatiotemporal regularities was completely dependent on intertrial priming (i.e., the regularities being present in the immediately preceding trial). The experimental task is depicted in Figure 5a. If short-term priming effects are necessary, then any advantage for predictable over unpredictable events should decline after a completely unpredictable trial (see hypothetical results in Figure 5b).[Fig fig5]

#### Behavioral Analysis

In line with our experimental manipulation, we included predictability, previous trial type, and their interaction as parameters in the model. By necessity, the first trial in each block and the completely random trials were not included in the analysis. The random-effects structure for the GLMM contained the participants’ intercepts as well as by-participant slopes for predictability and target order. This model was also optimal for the LMM. Significant interactions between predictability and trial type were broken down by defining difference contrasts to model the two critical comparisons (repeat vs. Nonrepeat trials for predictable and unpredictable targets).

### Results and Interim Discussion

Results are summarized in Figure 5c–f and showed that the effects of predictability were again preserved, with better performance for predictable targets even after fully random trials.

#### Accuracy

We found a significant effect of predictability on accuracy (Figure 5c; β = .20, *SE* = .02, *z* = 8.252, *p* < .001) and a significant effect of target order (Figure 5d; β = −.16, *SE* = .02, *z* = −7.45, *p* < .001). Observers were more accurate for predictable and early targets. We once again did not find a significant interaction between predictability and target order (β = .006, *SE* = .01, *z* = .43, *p* = .67). Moreover, we found no effect of the previous trial type (β = .02, *SE* = .02, *z* = 1.34, *p* = .18), and the previous trial type did not interact significantly with predictability (β = .02, *SE* = .02, *z* = 1.04, *p* = .30), indicating that there was no significant diminution in the predictability effect immediately following a fully random trial, in which all targets were unpredictable.

#### RTs

The equivalent analysis for RTs again showed faster responses for predictable targets (Figure 5e: β = −.015, *SE* = .006, *t* = −2.26, *p* = .03) and faster responses for early targets (Figure 5f: β = .11, *SE* = .007, *t* = 15.51, *p* < .001), yet no interaction (β = .006, *SE* = .003, *t* = 1.61, *p* = .11). There was also no effect of the previous trial type when considering RTs (β = −.005, *SE* = .004, *t* = −1.11, *p* = .27), and the previous trial type did not interact significantly with the predictability effect (β =.007, *SE* = .004, *t* = 1.61, *p* = .11).

## Experiment 4: Trial-Wise Priming Contributes to Behavioral Benefits

**Figure 6 fig6:**
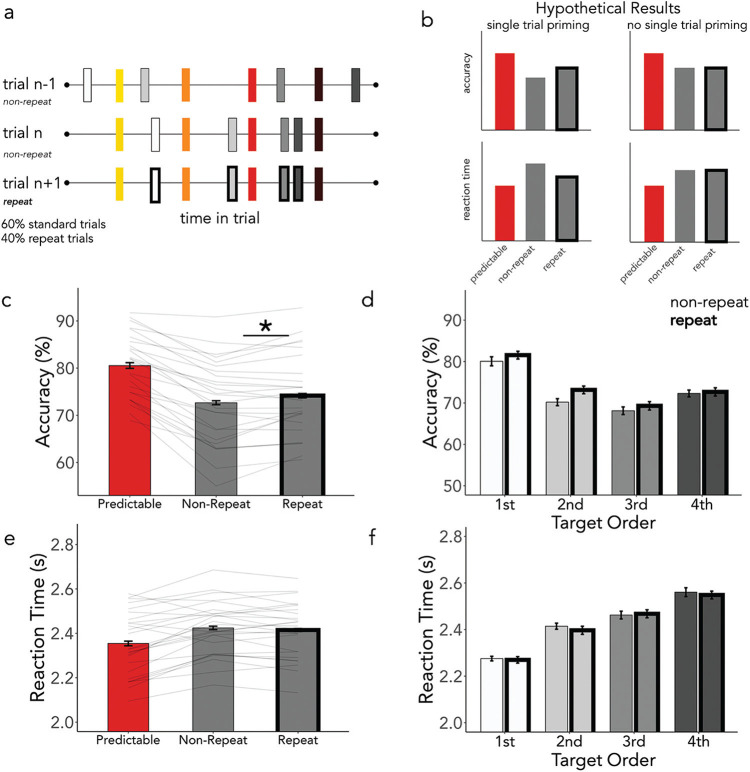
Trial-Wise Priming Contributes to Behavioral Benefits *Note*. (a) Depiction of a trial schematic for Experiment 4. Forty percent of trials were repeated such that the timings and quadrants of the unpredictable targets were the same as in the previous trial. (b) Depicted are hypothetical results supporting single-trial priming effects (left) and without evidence for single-trial priming effects (right). It is of note that if we find evidence to support the notion of single trial priming this should manifest in better performance in the repeat vs. nonrepeat trials. (c) Accuracy measures for predictable targets (in all trials) and the unpredictable targets in the nonrepeat vs. repeat trials. (d) Here we see the effect of repeating a single trial over the course of a trial; (e) compares reaction times for predictable targets, nonrepeat trials, and repeat trials. In (f) we see this effect across a trial. Asterisks indicate a *p*-value < .05. See the online article for the color version of this figure.

Complementing Experiment 3, in Experiment 4, we sought to assess the extent to which performance could also benefit from short-term single-trial priming effects. In Experiment 3, we found no significant diminution of the predictability effect in trials preceded by a random versus standard trial. Behavioral facilitation by spatiotemporal regularities was therefore resistant to interference of previous trials with a novel spatiotemporal pattern of targets. Nevertheless, it remains possible that short-term, single-trial priming effects may still also contribute to behavioral guidance. Specifically, in Experiment 4 we tested whether a single repetition of spatiotemporal target dynamics was sufficient to facilitate performance. Here we again introduced a new trial type. In repeat trials, all targets (including those that are overall unpredictable over the course of the experiment) appeared at the same time and quadrant as in the previous trial. If observers can benefit from a single repetition of spatiotemporal target information, we should find benefits for the normally unpredictable targets on repeat trials versus standard trials (see hypothetical results in Figure 6b).[Fig fig6]

### Method

#### Participants

In this experiment we tested the same group as in Experiment 3.

#### Apparatus

Again, participants completed the experiment in a group testing room with a capacity of 20 people, although no more than 12 were tested at once. Participants each sat approximately 50 cm from the monitor (Dell U2312HM Monitor, 1,920 × 1,080 resolution; refresh rate 60 Hz). The experimental script was again generated using the Psychophysics Toolbox (Brainard, 1997) on MATLAB (version 2014b, Mathworks, Natick, MA).

#### Stimuli

The stimuli were the same as in Experiments 2 and 3.

#### Procedure

We tested the participants in the same room and during the same session as in Experiment 3. The order of the two tasks (3 and 4) was counterbalanced.

In this experiment only 60% of the trials were standard “nonrepeat trials” (as described in Experiment 1). The remaining 40% of the trials were “repeat trials,” in which all of the target timings and quadrants from the previous trial repeated. That is, all eight targets had the same spatiotemporal dynamics as the previous trial. Although this was always the case for predictable targets, in repeat trials, what had been the four unpredictable targets on the previous trial now repeated their timing and quadrant from that trial. As such, performance on these one-time repetition targets would reveal if a single repetition of spatiotemporal information was enough to trigger a behavioral benefit. It is important to point out that although the repeated trials maintained the exact timing and quadrants from the previous trial, the exact location within the quadrant was random. Trial order was arranged such that repeat trials were always followed by standard trials. This ensured any effects were attributable to priming across single trials only. The experimental task outline is depicted in Figure 6a.

#### Behavioral Analysis

In Experiment 4, trial type and its interaction with predictability were included as predictors in the model. The random-effects structure for the GLMM contained the participants’ intercepts as well as by-participant slopes for predictability and target order. This model was also optimal for the LMM.

### Results and Interim Discussion

Results are shown in Figures 6c–f and showed that even a single repetition could improve accuracy performance though we did not find an effect of repetition on RT.

#### Accuracy

We once again found significantly higher accuracy for predictable compared to unpredictable targets (Figure 6c; β = .20, *SE* = .01, *z* = 10.96, *p* < .001) and a significant effect of target order, with early targets being detected more frequently (Figure 6d), (β = −.13, *SE* = .02, *z* = −8.25, *p* < .001). There was no interaction between predictability and target order (β = .006, *SE* = .01, *z* = .60, *p* = .55). Although, we did not find a significant effect of the trial repetition (β = −.004, *SE* = .01, *z* = −.33, *p* = .74), this factor interacted significantly with predictability (β = .03, *SE* = .01, *z* = 2.95, *p* = .003). Planned comparisons revealed that the “unpredictable” targets were found significantly more often in the repeat trials compared to the nonrepeat, standard trials (β = .08, *SE* = .03, *z* = 2.44, *p* = .01.

#### RTs

We found a main effect of predictability such that predictable targets were found faster than unpredictable targets (Figure 6e; β = −.03, *SE* = .006, *t* = -5.37, *p* < .001). Additionally, early targets were found faster (Figure 6f; β = .11, *SE* = .006, *t* = 18.46, *p* < .001). A significant interaction (β = .02, *SE* = .002, *t* = 7.57, *p* < .001) indicated a steeper slope of improvement in reaction times for predictable, compared to unpredictable targets—although, numerically, predictable targets were found faster than unpredictable targets throughout the trial. We found no effect of trial repetition on RTs (β = .004, *SE* = .003, *t* = 1.24, *p* = .21), and trial repetition did not interact significantly with the predictability effect (β = −.0005; *SE* = .003, *t* = −.16, *p* = .87).

## General Discussion

In four experiments, participants searched for targets among distractors in a novel dynamic visual-search task. Participants were more accurate and faster at detecting targets when they were predictable in their temporal onset and quadrant location. Experiment 1 established the basic effect in behavioral and eye-movement data. Experiment 2 showed that the effects were due to predictability of the specific temporal sequence of targets rather than just their temporal order or a rhythmic pattern. In Experiments 3 and 4 we showed that these behavioral benefits are likely attributable to the guidance of attention from memories at more than one time scale. In Experiment 3 we found that the predictability effect persists even after a completely random trial. This suggests that these benefits cannot be completely explained by single-trial priming (STM) and that a longer-term memory must be involved in order to maintain the regularities over several trials without any deficits. Even so, in Experiment 4, we found that single-trial priming effects do contribute to the overall effect since a single repeated trial was enough to trigger benefits in accuracy. Most likely, STM contributes to the learning of regularities, which, once learned, are held in a more robust longer-term store.

Models of visual search have evolved in their consideration of how attention may be allocated during search. Treisman’s Feature Integration Theory (Treisman & Gelade, 1980) was one of the first models to consider a serial deployment of attention over time. Eventually, models began to consider what information could be used to guide attention in a static scene (Egeth et al., 1984; Wolfe, 1994; Wolfe et al., 1989). Modern models pose that bottom-up saliency is integrate with information from the attentional template (a priori information that can guide search) in a continuously evolving priority map (Wolfe, 2021). However, these models have thus far not considered how temporal regularities within our environment may be additionally incorporated into attentional templates. Learning temporal predictions in dynamic contexts is particularly challenging since it involves the abstraction of temporal regularities from local temporal associations within the flow of information. Our results convincingly show that such the temporal regularities are learned and incorporated into dynamic priority maps that help guide attention, at least when combined with spatial regularities. Future models of visual search should therefore include consideration of the temporal dimension as a critical source of information to our attention system. Future experimentation will help specify the conditions under which temporal regularities support the guidance of attention and discover the psychological and neural mechanisms involved.

In the current task, each trial spanned several seconds and required multiple responses, allowing us to monitor the guidance of spatial selection over time. In designing the task, special consideration was given to the dynamics of the displays to minimize exogenous factors. Namely, targets appeared and disappeared slowly from the display, such that attention was not captured by the sudden onset of any event, but rather revealed the guidance by top-down predictive signals that changed dynamically over time. Nevertheless, interestingly, we consistently found a main effect of target order on our performance metrics. Performance was lowest when searching for the third target and improved again at the end. This U-shaped curve may reflect a combination of multiple factors linked to the dynamic and extended nature of the task. For example, performance may have benefited from slightly fewer competing distractors during the final time window in which no new distractors could fade in. Manipulating the frequency, timing and spatial distribution of distractors within the dynamic displays should prove an interesting avenue for future experimental research into how spatial-temporal predictions help overcome competition. In addition, the extended nature of the trial may also reveal natural intrinsic fluctuations in attention or arousal over time (Shalev, Bauer, et al., 2019). Additional recordings of brain and physiological activity (e.g., pupil diameter and blink rates) may provide traction into this interesting set of questions.

In the current work we have shown that spatiotemporal information about a target can be used to guide behavior. This was reflected not only in performance measures such as accuracy and reaction times, but also in eye movements being proactively guided to predictable compared to unpredictable targets. The necessary and sufficient conditions for learning and utilizing these spatial temporal regularities are not fully addressed by our current experimental design. For example, in future studies we intend to probe whether the effects are dependent on responding overtly to targets (action). Attention and action are tightly coupled (Heuer et al., 2020; Olivers & Roelfsema, 2020; van Ede, 2020); and it remains unclear whether these regularities can be learned or utilized in their absence (Shalev, Nobre, et al., 2019). Ultimately, this experimental framework could be applied to understand the contributions of and interactions among regularities along various stimulus dimensions, including their action associations. For now, the necessary building blocks for the effects remain an important topic for investigation.

Moreover, whereas we demonstrated clear and consistent effects on accuracy and response times, we did not explore the full gamut of potential performance benefits. it remains unclear, for example, whether spatiotemporal predictions can shift observers’ criterion in dynamic visual search. We relied on targets that were readily distinguished from the distractors (vertical lines vs. lines tilted between 80° and 100°). We do not expect that observers would have often misreported distractors, but we did not record these possible errors. One could imagine that the increase in performance related to the predictable targets is in part related to dynamic shifts in criterion over the course of the trial. If this were the case, in addition to the high accuracy related to finding more predictable targets, we would also expect higher false alarms at the moments near the expect target onset. It is a limitation of the current work that we cannot speak to shifts in criterion directly, but future studies can examine these systematically, including by varying target–distractor similarity.

In Experiments 3 and 4 we arranged “random” (Experiment 3) and “repeat” (Experiment 4) trials such that they were always preceded by a “standard” trial. This was considered an important control for specifically examining short-term influences from one individual trial on the next. In Experiment 3, we were interested in whether an interruption of regularities in a single random trial would erode benefits in performance to predictable targets in subsequent trials. We did not find such an effect, suggesting that behavioral benefits were not solely attributable to single trial priming effects. It remains unclear from the current work how several random trials in a row may diminish the effects of predictions in subsequent trials. In Experiment 4 we posed a complementary question: whether single-trial priming effects were sufficient to elicit benefits. We found that a single repetition was indeed sufficient to confer better accuracy for finding previously unpredictable targets. No benefit occurred for in reaction times, and we believe this may point to an interesting functional dissociation between accuracy and speed, which should be examined in future studies.

Compared with many studies considering how memory guides spatial attention in static displays (Chun & Jiang, 1998; Geng & Behrmann, 2002; Hutchinson & Turk-Browne, 2012; Li & Theeuwes, 2020; Summerfield et al., 2006), there has been less work on the spatial guidance of attention in environments that unfold over time. Cravo and colleagues (2017) demonstrated that observers are able to utilize temporal intervals associated with specific contexts to guide attention—although in a task that utilized static displays with sudden target onsets. Muhl-Richardson, Cornes, et al. (2018) and Muhl-Richardson, Godwin, et al. (2018) showed that observers prioritized distractors in a dynamically changing display when these distractors had a high probability of soon becoming targets. Other studies showed how repeating sequences can inform attentional guidance (Boettcher et al., 2020; Heideman et al., 2018; Li & Theeuwes, 2020; Nobre & O’Reilly, 2004; O’Reilly et al., 2008; Zhao et al., 2013). Here we demonstrate for the first time that memory-based spatiotemporal predictions can drive behavior in an extended dynamic task when facing multiple competing signals (i.e., distractors and other targets). Importantly, we find a behavioral benefit of predictions when the spatiotemporal relationship was a high-level representation that was abstracted beyond simple temporal associations between successive pairs of stimuli.

We demonstrated that spatial and temporal guidance of attention can work together to benefit performance within extended dynamic contexts. Our results extend findings showing strong interactions between spatially and temporally informative cues in guiding attention (Doherty et al., 2005; Heideman et al., 2018; Nobre & Rohenkohl, 2014; Rohenkohl et al., 2014) by showing that spatiotemporal predictions go beyond simple associations between two discrete stimuli. In our task, spatiotemporal predictions were driven by implicit task regularities. These in turn led to proactive allocation of spatial attention as demonstrated through eye movements: observers fixated the relevant quadrant earlier when targets were predictable and ahead of their manual responses. The current work provides further evidence for temporal orienting of attention, here in a dynamic context even with a certain amount of spatial uncertainty. Therefore, we have replicated this previously found synergistic relationship between temporal and spatial attention. Within our task we did not seek to fractionate the individual contributions of spatial and temporal predictions within this dynamic search context since both types of regularities covaried in our task. Future studies may wish to separate the individual contributions of these mechanisms as well as explore the potential interactions between these predictive sources.

In the current work, we have introduced a new perspective for considering spatial attention in visual search by including time as an informative dimension. Through this manipulation, we have found that the spatial distribution of attention is allocated flexibly on the basis of temporal predictions. Our task can be extended in several directions to explore whether additional sources of guidance also evolve with time. For instance, it may be interesting to consider whether various features can be prioritized dynamically; for example, whether knowing that a colored target is likely to emerge at a predictable time, without knowing where, can also benefit performance. Additionally, the new experimental framework can be used to characterize the precision of temporal and spatial predictions, independently, by presenting targets across a range of moments in time or locations in space in order to manipulate degrees of temporal and spatial competition.

In designing our stimulus arrays, we made some particular choices that may have contributed to the pattern and strength of our effects. Our stimulus displays contained four spatially distinct quadrants. The distinctiveness of the quadrants may have made it easier for observers to associate time and space. In past work it has been shown that observers are able to learn regularities that exist on a quadrant level (i.e., highly probable target quadrant) even when the quadrants were not visually obvious from the display (Jiang, 2018; Jiang, Swallow, & Rosenbaum, 2013; Jiang, Swallow, Rosenbaum, & Herzig, 2013). This suggests distinctiveness of the quadrants is not strictly necessary to learn these regularities, however, this remains untested and will be addressed by future work. The stimuli appeared and disappeared from the array gradually. This was done deliberately, to minimizes exogenous attraction of attention. While in the current investigation this was necessary in order to reduce the influence of bottom-up capture, this can be directly manipulated in the future.

We demonstrate that spatiotemporal regularities guide attention to the right place at the right time in complex visual search tasks. Memories from multiple timescales can support attentional guidance in these more naturalistic settings. Our simple—yet powerful—experimental framework promises to further the investigation into the dynamic factors guiding attention. Moving forward, time should be considered not only as a crucial dimension for understanding natural behavior, but also as powerful axes over which predictions may be formed.

## Supplementary Material

10.1037/xge0000901.supp
