# Supplementary material for: Right Place, Right Time: Spatiotemporal Predictions Guide Attention in Dynamic Visual Search
Source: J Exp Psychol Gen. 2021 Nov 29;151(2):348–62. doi: 10.1037/xge0000901 (PMC8920297; doi:10.1037/xge0000901)
Supplement: Supplementary file 1 [file xge0000901.pdf]

Supplementary Tables

	Target Predictability				Target Order				Target Predictability:Target Order				Trial Type				Target Predictability:Trial Type			
**Accuracy**	df	F	p	η^2_G	df	F	p	η^2_G	df	F	p	η^2_G	df	F	p	η^2_G	df	F	p	η^2_G
**Experiment 1**	1,24	34.32	< .001 ***	0.08	3,72	16.77	< .001***	0.08	3,72	2.02	0.11	0.009								
**Experiment 2**	.73,16.8	47.2	< .001 ***	0.13	3,69	28.37	< .001***	0.14	3,69	0.24	0.87	0.001								
**Experiment 3**	1,25	58.67	< .001***	0.11	2.25,56.3	47.51	< .001***	0.2	3,75	0.81	0.49	0.002	1,25	2.24	0.15	0.001	1,25	0.56	0.46	0.0007
**Experiment 4**	1,25	90	< .001***	0.14	3,75	66.3	< .001***	0.21	3,75	1.69	0.18	0.006	1,25	0.55	0.46	0.0002	1,25	10.23	0.003 **	0.004

Table 1: ANOVA results for accuracy in all four experiments. * p < .05, ** p < .01, *** p < .001.

	Target Predictability				Target Order				Target Predictability:Target Order				Trial Type				Target Predictability:Trial Type			
**RT**	df	F	p	η^2_G	df	F	p	η^2_G	df	F	p	η^2_G	df	F	p	η^2_G	df	F	p	η^2_G
**Experiment 1**	1,24	152.7	< .001 ***	0.06	3,72	64.5	< .001 ***	0.1	2.00,48.1	2.92	0.06	0.003								
**Experiment 2**	1,23	20.18	< .001 ***	0.013	3,69	78.68	< .001 ***	0.21	3,69	1.21	0.31	0.004								
**Experiment 3**	1,25	3,12	0.08	0.007	3,75	122.96	< .001***	0.34	3,75	1.24	0.3	0.003	1,25	0.9	0.35	0.0008	1,25	1..77	0.2	0.002
**Experiment 4**	1,25	26.6	< .001***	0.039	3,75	204.6	< .001***	0.41	1.73,43.36	9.3	< .001 ***	0.033	1,25	1.18	0.29	0.0005	1,25	0.54	0.97	<.0001

Table 1: ANOVA results for RT in all four experiments. * p < .05, ** p < .01, *** p < .001.

Supplementary Figure

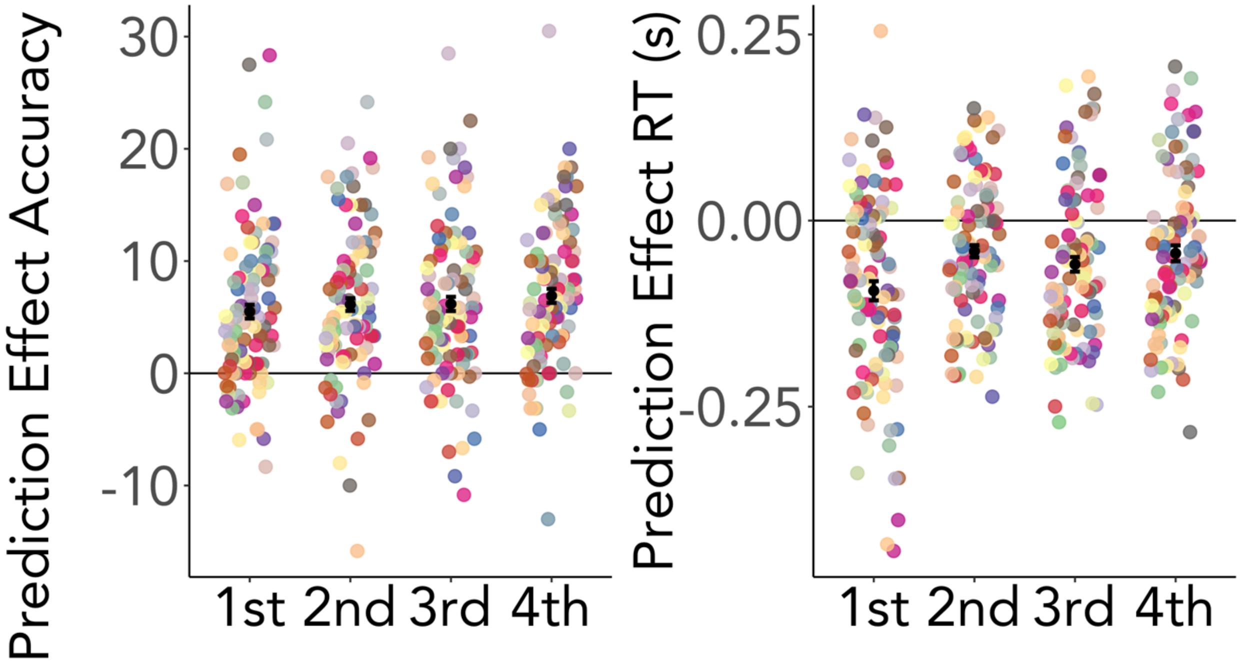

Supplementary Figure 1: The prediction effect (predictable – unpredictable) for each subject at each time bin within the trial. Means and standard error are represented in black. The individual subjects are represented by the different colours. Panel a) shows the accuracy effect whereas panel b) shows the RT effect. As can be seen here, most subjects show the effect in most blocks.
